# Visual Processing in Infants with Tuberous Sclerosis Complex

**DOI:** 10.15844/pedneurbriefs-30-2-2

**Published:** 2016-02

**Authors:** Tracy S. Gertler, Srishti Nangia

**Affiliations:** 1 Division of Neurology, Ann & Robert H. Lurie Children's Hospital of Chicago, Chicago, IL; 2 Departments of Pediatrics and Neurology, Northwestern University Feinberg School of Medicine, Chicago, IL

**Keywords:** VEP, Tuberous Sclerosis Complex, Visual Processing

## Abstract

Investigators from Harvard University and UCLA have reported that despite evidence of structural abnormalities in the visual pathway of animal models and children with tuberous sclerosis complex (TSC), visual evoked potentials (VEPs) in 12-month old children with TSC compared to an age-matched control group are not significantly altered.

Investigators from Harvard University and UCLA have reported that despite evidence of structural abnormalities in the visual pathway of animal models and children with tuberous sclerosis complex (TSC), visual evoked potentials (VEPs) in 12-month old children with TSC compared to an age-matched control group are not significantly altered. This study is motivated by the high rates of autism spectrum disorder in children with TSC, which the authors posit may be due to delayed visual processing.

This multisite, longitudinal case-control study compares 16 infants with TSC (including a majority with retinal hamartomas, occipital tubers, and intractable epilepsy on vigabatrin) to 18 “typically-developing infants.” Infants were presented a black and white phase-reversing checkerboard during which visual fixation was tracked, and 50-100 trials were recorded. Peak amplitude and latency of the N1, P1, and N2, the primary components of the visual evoked potential, were measured and were found to be not significantly different in a non-parametric analysis. [1]

COMMENTARY. This paper seeks to identify an early and easily-tested surrogate for neurodevelopmental impairment in children with TSC, as increased rates of autism in the TSC population suggest the increased prevalence in this population might warrant early therapeutic interventions in the future if possible. In Drosophila lacking *TSC1* in the retina, both the photoreceptor layer itself and the axon bundle are severely disrupted [2]; similarly, *TSC2* gene mutation leads to enlarged photoreceptor and support cell units (ommatidia) with failure to prune synaptic connections [3]. Further study of heterozygous *TSC2* mice has implicated ephrin-EphA receptor signaling in abnormal development of retinogeniculate connections [4], presumably through activation of the mTOR pathway via ERK1/2 inhibition and thus *Tsc2* activation. When taken in the context with studies of patients with TSC showing that a delay in nonverbal ability, primarily visual processing, was an early biomarker for cognitive impairment [5], the authors’ hypothesis that visual connectivity is altered early in life in TSC is logical and warrants further testing.

As measurement of a visual evoked potential (VEP) surveys the visual pathway from retina to primary visual cortex, any aberration in synaptic connectivity along this pathway would be expected to impact latency or amplitude in a population-based fashion. VEPs would also prove useful in a clinical setting, as infants with limited ability to cooperate could be tested at different age intervals. However, despite known MRI abnormalities in the occipital region and concurrent diagnosis and treatment of an epileptic encephalopathy, infants in this study did not manifest any systematic difference in VEPs. Potential explanations for this interesting yet unexpected negative finding include the level of visual processing studied - for example, while a previous study noted specifically a deficit in face processing in individuals with TSC [6], this higher-order deficit would not be obvious on VEP. Replication of these results at other developmental stages, for example as autistic behaviors or learning disabilities become more clinically apparent, would also provide valuable information. Nevertheless, the idea that the minimal visual substrate is intact in symptomatic TSC infants represents an important observation in understanding the nature of deficient visual processing.
